# Mechanical Thrombus Aspiration for a Young STEMI Patient With Plaque Erosion: Time for a Stent‐Free Approach?

**DOI:** 10.1002/ccd.70726

**Published:** 2026-07-03

**Authors:** Domenico Simone Castiello, Francesco Greco, Antonio Curcio, Alberto Polimeni

**Affiliations:** ^1^ Department of Pharmacy, Health and Nutritional Sciences University of Calabria Rende Italy; ^2^ Division of Cardiology Annunziata Hospital Cosenza Italy; ^3^ Department of Advanced Biomedical Sciences University Federico II Naples Italy; ^4^ Division of Interventional Cardiology Annunziata Hospital Cosenza Italy

**Keywords:** acute coronary syndrome, coronary mechanical thrombus aspiration, intracoronary imaging, plaque erosion, ST‐elevation myocardial infarction

## Abstract

A 34‐year‐old man with no cardiovascular history presented with anterior ST‐elevation myocardial infarction and a large thrombotic occlusion of the mid left anterior descending artery. Following potent antithrombotic therapy and mechanical thrombus aspiration, angiography revealed vessel patency without residual significant stenosis, allowing a deferred stent strategy. Subsequent optical coherence tomography confirmed definite plaque erosion, supporting conservative management with dual antiplatelet and intensive lipid‐lowering therapy. At 1‐year follow‐up, the patient remained asymptomatic with no adverse events, underscoring the feasibility of an imaging‐guided, stent‐free approach in selected young patients with erosion‐related acute coronary syndromes.

## 1 Introduction

Plaque erosion is increasingly recognized as a distinct pathophysiological substrate of acute coronary syndromes (ACS), characterized by endothelial denudation and thrombus formation over an intact fibrous cap, in the absence of frank plaque rupture [1]. Unlike rupture‐prone lesions, erosions are often associated with less lipid burden, preserved fibrous architecture, and a higher prevalence in younger patients and smokers. Contemporary intracoronary imaging studies suggest that plaque erosion accounts for approximately 25%–35% of ST‐elevation myocardial infarction (STEMI) presentations, particularly in younger patients [2]. These mechanistic insights have important therapeutic implications: in selected patients with preserved vessel architecture and no critical residual stenosis after thrombus resolution, a stent‐free strategy guided by intracoronary imaging may be feasible. Intensive antithrombotic therapy combined with aggressive secondary prevention can promote plaque healing while avoiding the long‐term risks associated with a permanent metallic stent [3]. In this case report, we describe a young male patient presenting with anterior STEMI and complete thrombotic occlusion of the mid left anterior descending (LAD) artery due to plaque erosion, conservatively managed with a stent‐free approach combining medical therapy and mechanical coronary thrombus aspiration.

## 2 Case Presentation

A 34‐year‐old man with a negative family history for coronary artery disease (CAD) and without known major cardiovascular events or pharmacological therapies presented to the emergency department with severe chest pain and diaphoresis.

The electrocardiogram revealed ST‐segment elevation in precordial leads, whereas the urgent transthoracic echocardiography showed a mildly reduced left ventricular systolic function of 45%, with evidence of septal and apical akinesia. A diagnosis of anterior STEMI was made, and the patient was urgently transferred to the catheterization laboratory.

**Figure 1:**
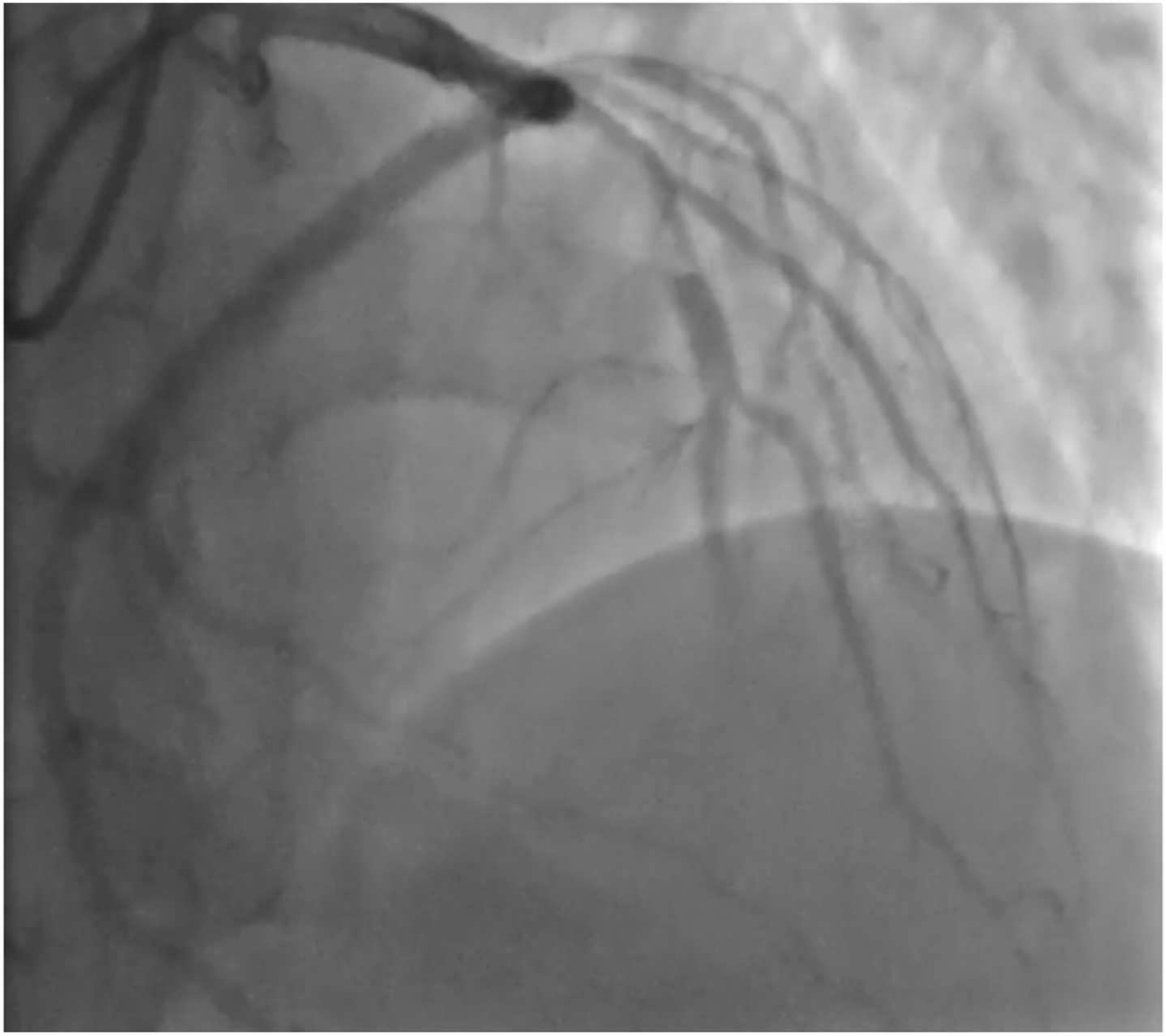
Angiographic evidence of a subocclusive stenosis in the mid left anterior descending coronary artery, followed by thrombotic occlusion with a high thrombus burden.

**Figure 2:**
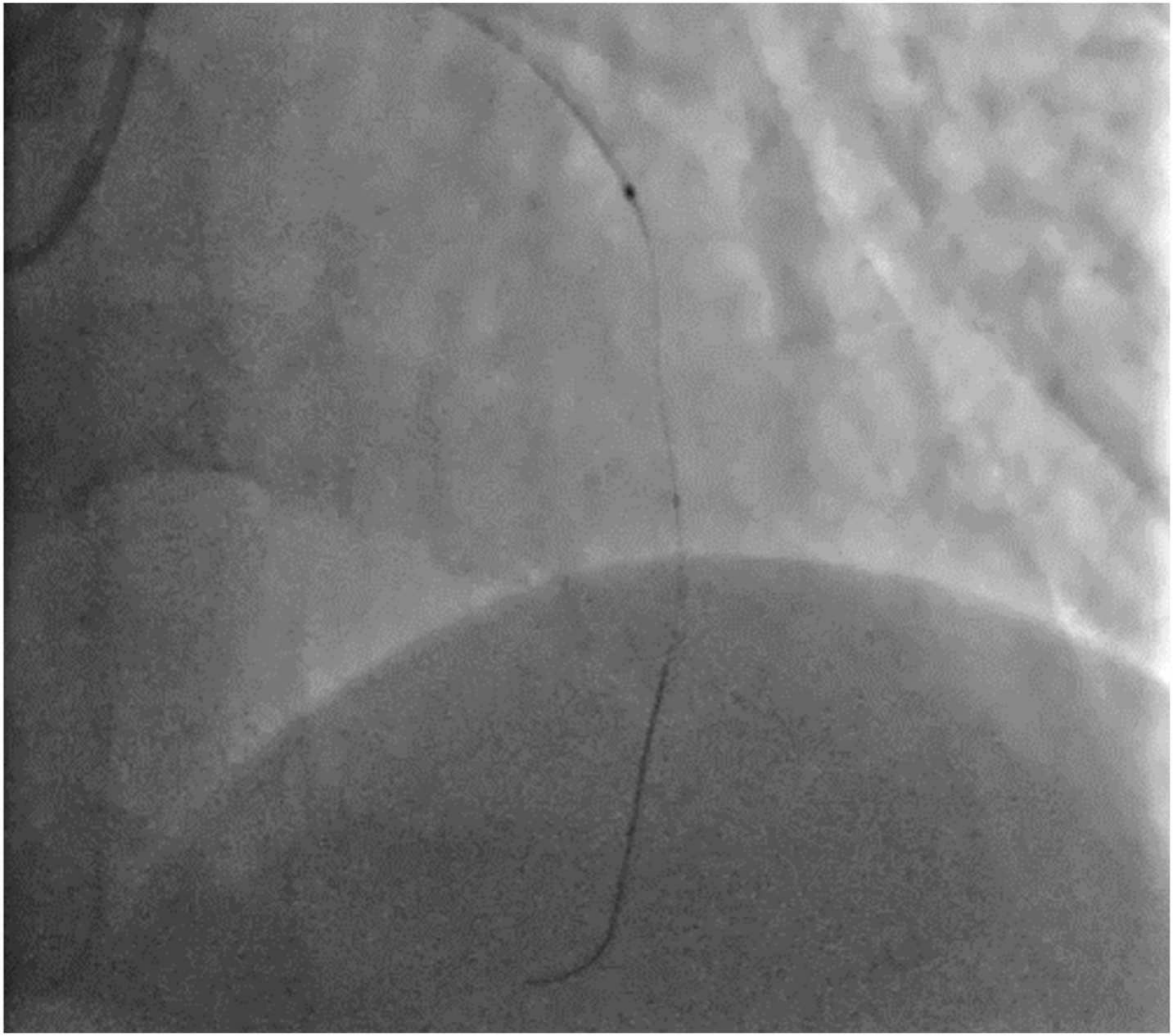
Mechanical coronary thrombus aspiration with CAT‐RX Penumbra (Indigo) device.

**Figure 3:**
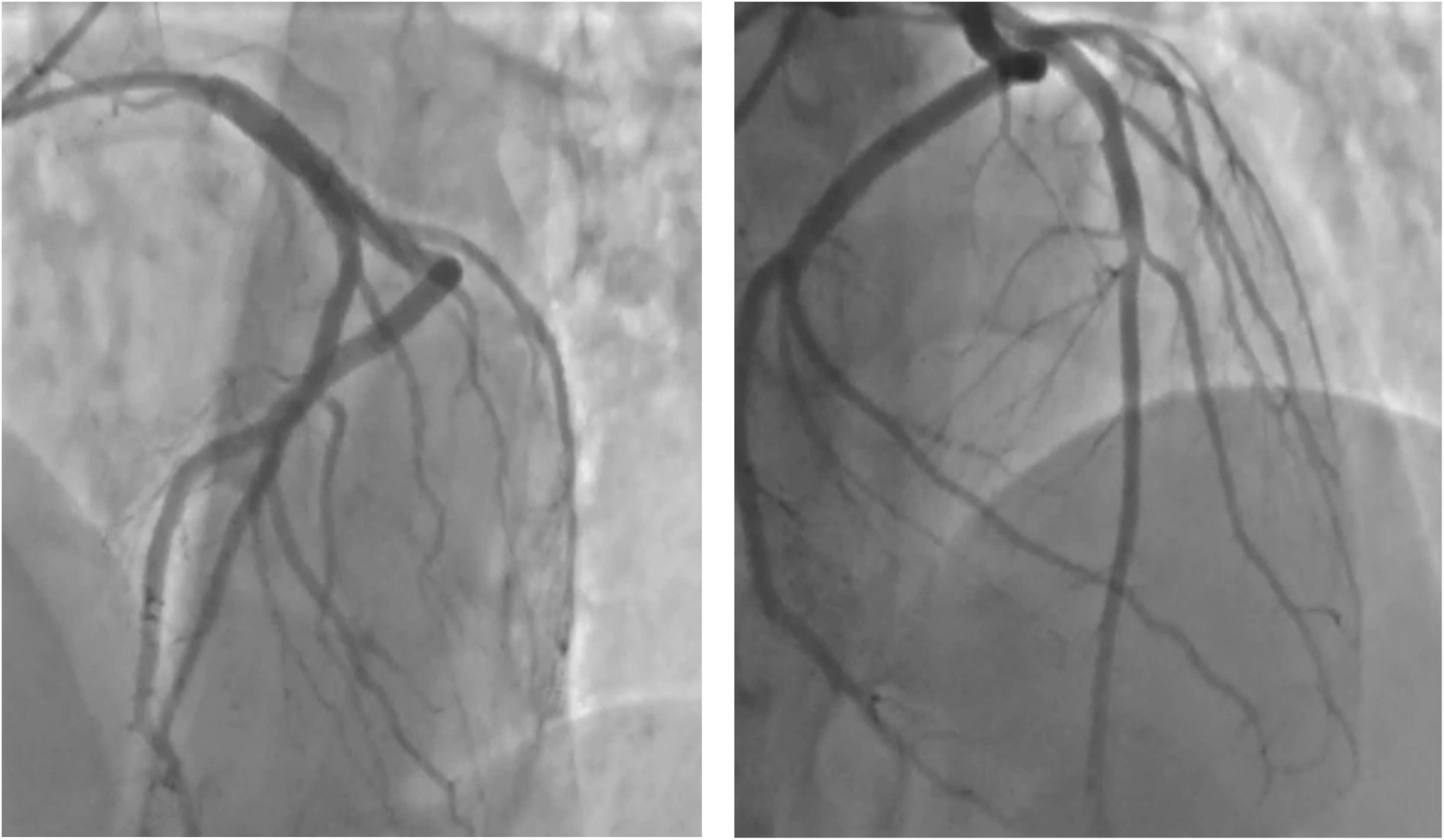
Restoration of TIMI 3 antegrade coronary flow following effective mechanical coronary thrombus aspiration of the left anterior descending coronary artery.

**Figure 4:**
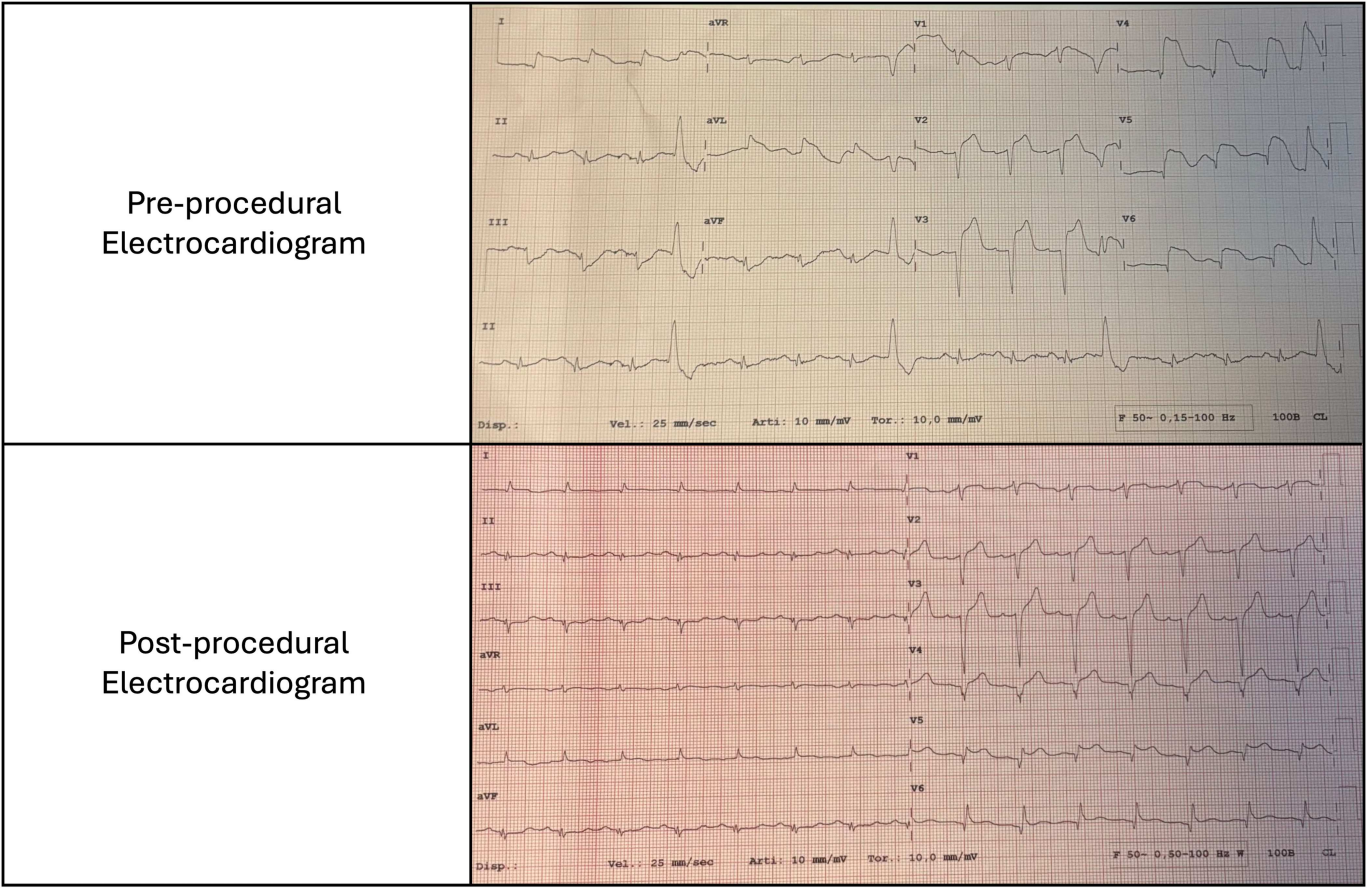
Representative pre‐procedural and post‐procedural electrocardiograms demonstrating marked reduction of anterior ST‐segment elevation following mechanical thrombus aspiration and restoration of Thrombolysis In Myocardial Infarction (TIMI) 3 coronary flow. [Color figure can be viewed at wileyonlinelibrary.com]

**Figure 5:**
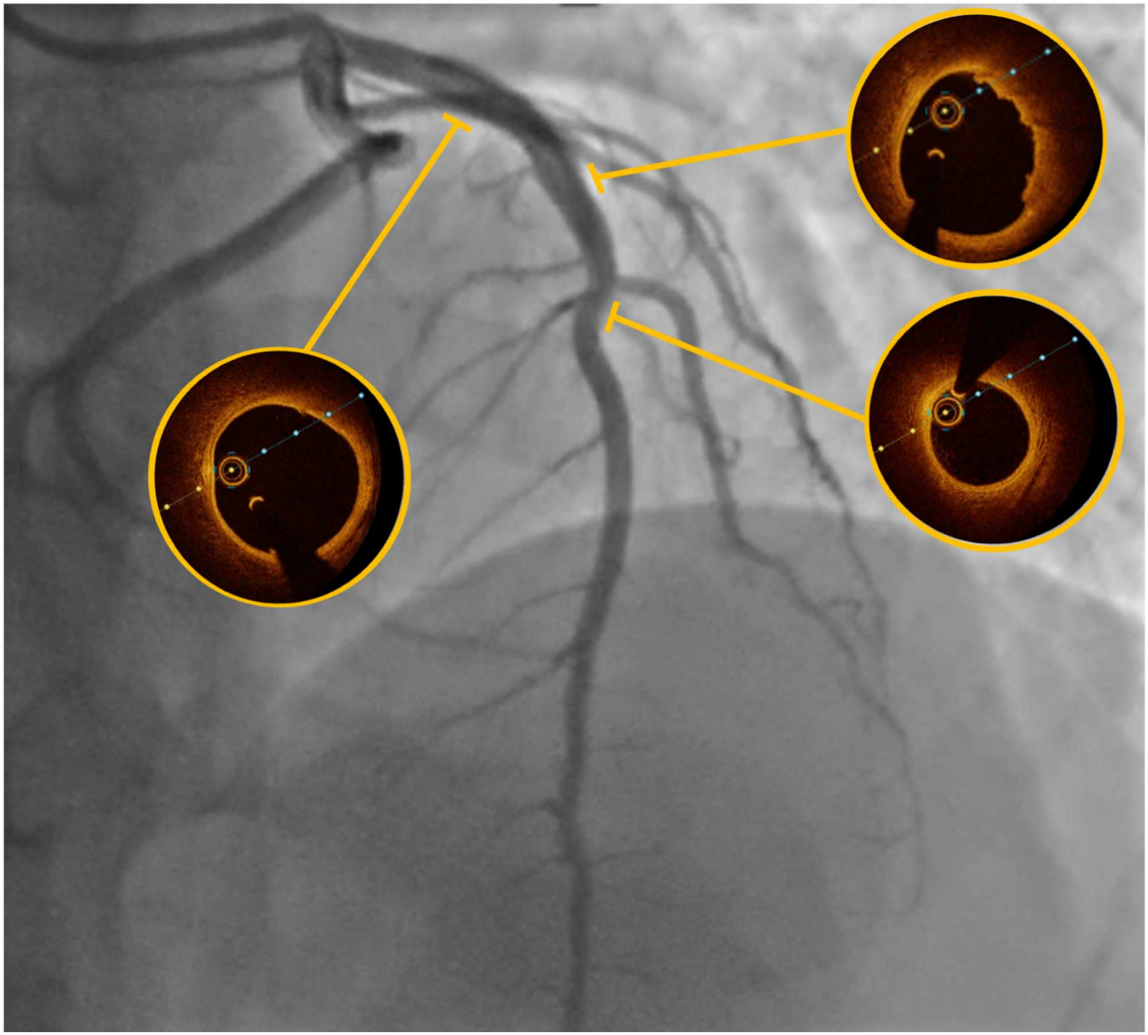
Optical coherence tomography (OCT) assessment on the left anterior descending artery, demonstrating plaque erosion in the mid segment. [Color figure can be viewed at wileyonlinelibrary.com]

**Figure 6:**
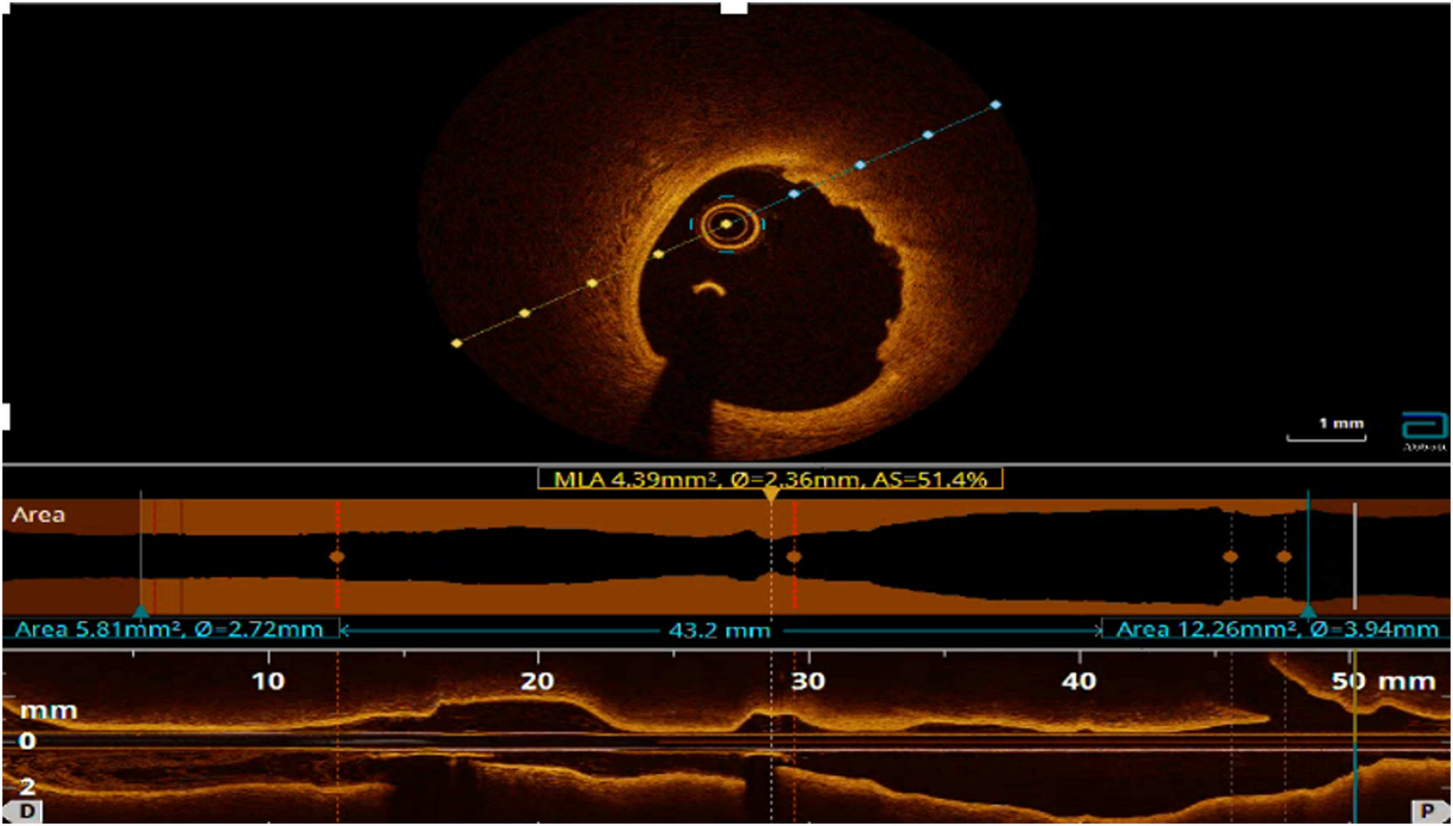
Optical coherence tomography (OCT) imaging demonstrating plaque erosion with adherent thrombotic foci overlying an intact fibrous cap in the mid‐segment of the left anterior descending coronary artery. [Color figure can be viewed at wileyonlinelibrary.com]

**Figure 7:**
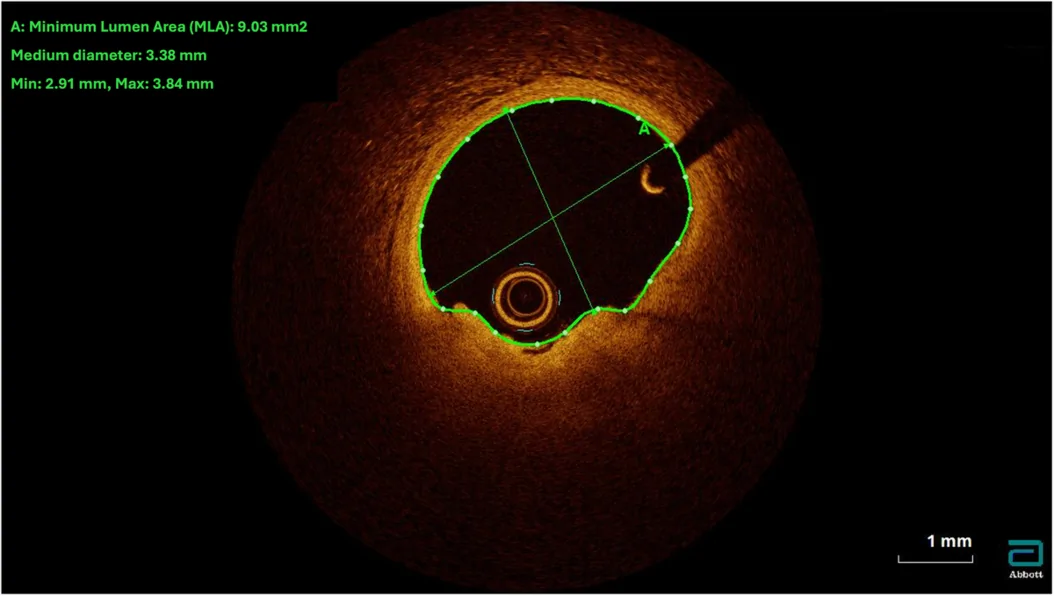
Optical coherence tomography (OCT) imaging demonstrating a large residual luminal area at the culprit site with a minimum lumen area (MLA) of 9.03 mm^2^. [Color figure can be viewed at wileyonlinelibrary.com]

**Figure 8:**
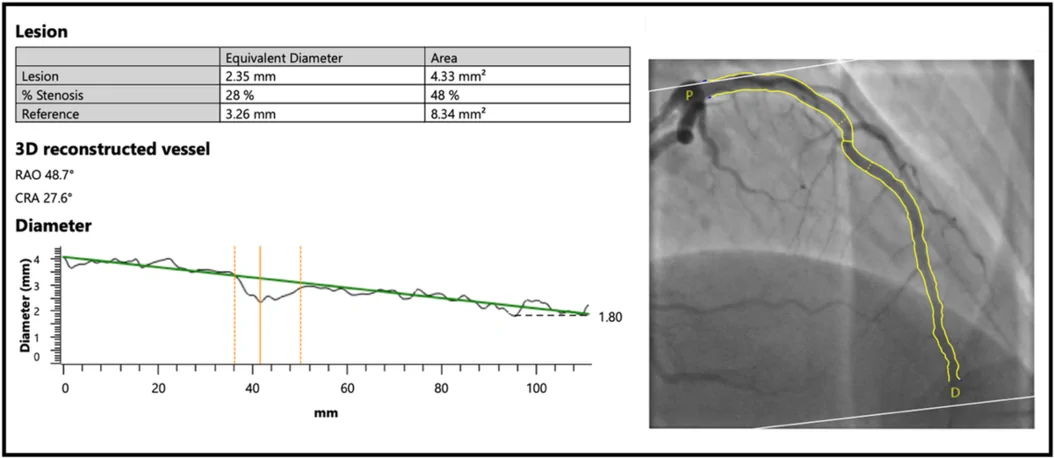
Lesion assessment with evidence of mild residual stenosis of 28% with quantitative coronary angiography (QCA). [Color figure can be viewed at wileyonlinelibrary.com]

The coronary angiography, performed through a left radial access using a 6 Fr Backup Left (BL) 3.5 guiding catheter, revealed a subocclusive stenosis at the mid LAD followed by a complete thrombotic occlusion with a Thrombolysis In Myocardial Infarction (TIMI) thrombus grade of 5 (Figure 1). Considering the large thrombotic burden observed, a potent antithrombotic therapy was initiated with intravenous unfractionated heparin (UFH) and intracoronary tirofiban. The patient had initially received 5000 U of UFH during ambulance transfer. Subsequently, after documentation of an activated clotting time (ACT) value of 140 s, an additional 5000 U of UFH was administered, achieving a final ACT of 250 s during the procedure. Intracoronary tirofiban was administered as a bolus of 25 mcg/kg followed by an intravenous infusion at a dose of 0.15 mcg/kg/min (then continued for 6 h in the intensive care unit) [4]. Furthermore, using a BMW Universal II coronary guidewire, mechanical coronary thrombus aspiration was performed using the CAT‐RX Penumbra (Indigo) device, with three aspiration passes over a total procedural aspiration time of approximately 40 s, aspirating nearly 70 cc of blood (Figure 2). This strategy resulted in complete thrombus removal with restoration of TIMI 3 flow and an estimated 100% angiographic improvement in vessel visualization. Only a minimal residual thrombotic burden with mild angiographic haziness persisted after the procedure, corresponding to a TIMI thrombus grade 1. Myocardial reperfusion was satisfactory, with a myocardial blush grade of 2 and no evidence of distal embolization or no‐reflow phenomenon. The final angiographic result showed a patent LAD, without significant residual stenosis (Figure 3). In addition, a significant electrocardiographic improvement was observed during the procedure, with 80% resolution of ST‐segment elevation (Figure 4). Therefore, no balloon dilatation or stent implantation was performed, and an intracoronary imaging assessment was scheduled several days later to clarify the underlying pathophysiological mechanism. The decision to defer OCT assessment was mainly guided by clinical safety considerations. Indeed, during the index procedure, the patient developed hypotension associated with mild pulmonary congestion/sub‐edema requiring stabilization and close monitoring in the coronary intensive care unit. Furthermore, the loading doses of aspirin (300 mg) and ticagrelor (180 mg) had been administered only approximately 30 min before coronary angiography, and therefore additional time was allowed to achieve a more complete pharmacodynamic antiplatelet effect. The OCT reassessment was subsequently postponed for an additional 48 h because of a transient febrile episode associated with neutrophilic leucocytosis and increased C‐reactive protein levels, in order to perform the second invasive procedure under more stable clinical conditions. Accordingly, 5 days later, the patient underwent optical coherence tomography (OCT) assessment. A final diagnosis of definite plaque erosion was established according to the standardized OCT criteria proposed by Jia et al. defined by the presence of thrombus overlying a clearly visualized plaque with an intact fibrous cap, without evidence of cap disruption [1]. In the present case, OCT showed residual intraluminal thrombus spots adherent to the luminal surface of the culprit plaque at the mid LAD, while the underlying fibrous cap appeared continuous and intact (Figures 5, 6). Plaque rupture was carefully excluded because no fibrous cap discontinuity, intraplaque cavity formation, or communication between the vessel lumen and the underlying plaque core was observed. Moreover, no OCT features suggestive of a calcified nodule or other culprit morphologies were identified. The plaque erosion longitudinal extension measured approximately 7 mm, whereas the minimum lumen area (MLA) at the culprit site was 9.03 mm^2^ on OCT assessment (Figure 7). Importantly, a minimal residual stenosis of 28% was revealed through quantitative coronary angiography (QCA), while no flow‐limiting dissection nor vessel recoil were found, both angiographically and at intracoronary imaging reassessment (Figure 8). Based on these findings, considering the preserved vessel architecture, the absence of significant residual stenoses, the large residual luminal area, and the restoration of stable TIMI 3 coronary flow, the decision to pursue conservative management with a stent‐free approach was confirmed.

During the hospitalization, severe dyslipidemia was identified (LDL‐cholesterol: 213 mg/dL). The patient was a non‐smoker and had no previously known cardiovascular risk factors or family history of premature CAD. Considering the very young age and the markedly elevated LDL‐cholesterol levels, familial hypercholesterolemia was clinically suspected. However, subsequent genetic testing excluded pathogenic variants associated with familial hypercholesterolemia. Moreover, a complete thrombophilia screening and drug testing were performed, both yielding negative results. Given the presentation with ACS, the patient was considered at very high cardiovascular risk. Therefore, an intensive ‘fast‐track’ lipid‐lowering strategy was initiated, consisting of a high‐intensity statin (rosuvastatin 20 mg qd), ezetimibe 10 mg qd, and evolocumab 140 mg every 2 weeks, with the aim of rapidly achieving an LDL‐C level < 55 mg/dL [5, 6, 7]. Rosuvastatin 20 mg was preferred over maximal statin dosing in order to achieve an immediate and profound LDL‐cholesterol reduction while minimizing the potential risk of statin‐associated muscle symptoms, which could have negatively affected long‐term adherence in such a young patient. The clinical course was uneventful, and the patient was discharged on dual antiplatelet therapy (DAPT) with aspirin 100 mg qd and ticagrelor 90 mg bid. DAPT was continued for 12 months according to current European ACS guidelines recommendations and the 1‐year findings of the EROSION (Effective Anti‐Thrombotic Therapy Without Stenting: Intravascular OCT–Based Management in Plaque Erosion) trial, considering also the absence of high bleeding risk features despite the no‐stent strategy [5, 8]. Nevertheless, the optimal duration of DAPT in patients with OCT‐defined plaque erosion managed conservatively remains incompletely established.

**Figure 9:**
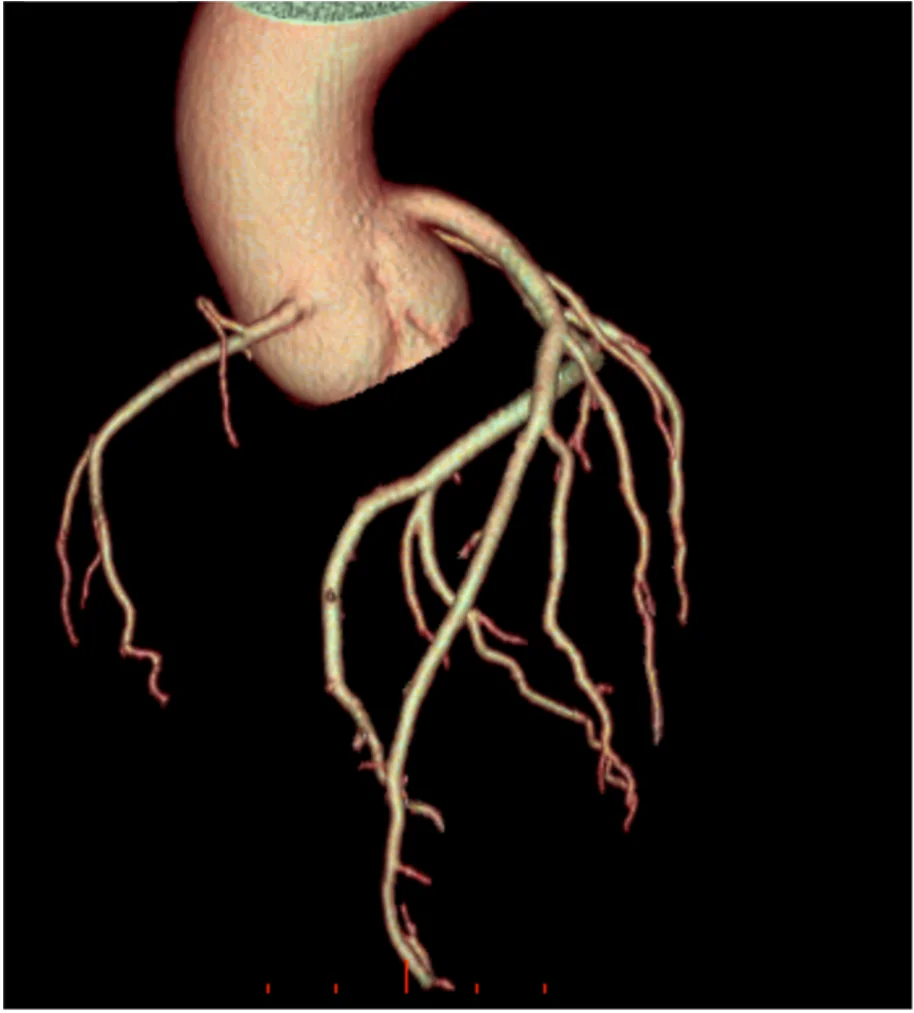
Coronary computed tomography angiography (CCTA) demonstrating epicardial coronary arteries free from significant stenosis at 1‐year follow‐up after the acute event. [Color figure can be viewed at wileyonlinelibrary.com]

During follow‐up, the patient showed excellent adherence to pharmacological therapy and lifestyle recommendations. At both 1‐month and 6‐month clinical follow‐up visits, he remained completely asymptomatic and free from adverse cardiovascular events. At the longest available follow‐up of 12 months, the patient continued to be asymptomatic, without recurrent ischemic events or need for rehospitalization. Echocardiographic reassessment demonstrated significant left ventricular recovery, with improvement of left ventricular ejection fraction from 50% at discharge to 58%, in the absence of residual regional wall motion abnormalities. In order to avoid an additional invasive coronary angiographic reassessment in this young patient, coronary computed tomography angiography (CCTA) was performed at 1 year and demonstrated patent epicardial coronary arteries without significant residual stenosis (Figure 9). LDL‐cholesterol levels decreased to 35 mg/dL at 12 months.

## 3 Conclusion

This clinical case supports the hypothesis that in patients with ACS caused by plaque erosion, antithrombotic therapy with a stent‐free approach may be a safe and effective option. Moreover, although large, randomized trials (namely, TAPAS [Thrombus Aspiration During Percutaneous Coronary Intervention in Acute Myocardial Infarction], TASTE [Thrombus Aspiration in STEMI in Scandinavia] and TOTAL [Trial of Routine Aspiration Thrombectomy With PCI Versus PCI Alone in Patients With STEMI] trials) failed to demonstrate a clinical benefit of routine thrombus aspiration during primary PCI [9, 10, 11], and current European Society of Cardiology guidelines do not recommend its routine use in STEMI patients [5], selected cases with exceptionally high thrombotic burden (TIMI thrombus grade ≥ 3) may still derive procedural benefit from this strategy [12]. In the present case, mechanical thrombus aspiration was performed in a highly selected clinical scenario characterized by TIMI thrombus grade 5, allowing effective thrombus removal, restoration of TIMI 3 flow, and subsequent intracoronary imaging‐guided conservative management without stent implantation. Finally, although delayed OCT assessment after intensive antithrombotic therapy represents a limitation of this case, this experience reinforces the paradigm that STEMI is not a single disease but a spectrum of pathophysiological mechanisms requiring individualized management. Especially for young patients, precision medicine guided by an accurate diagnostic assessment with intracoronary imaging can avoid unnecessary stent implantation and profoundly influence both immediate and long‐term outcomes (Central Illustration 1).

**Central Illustration 1:**
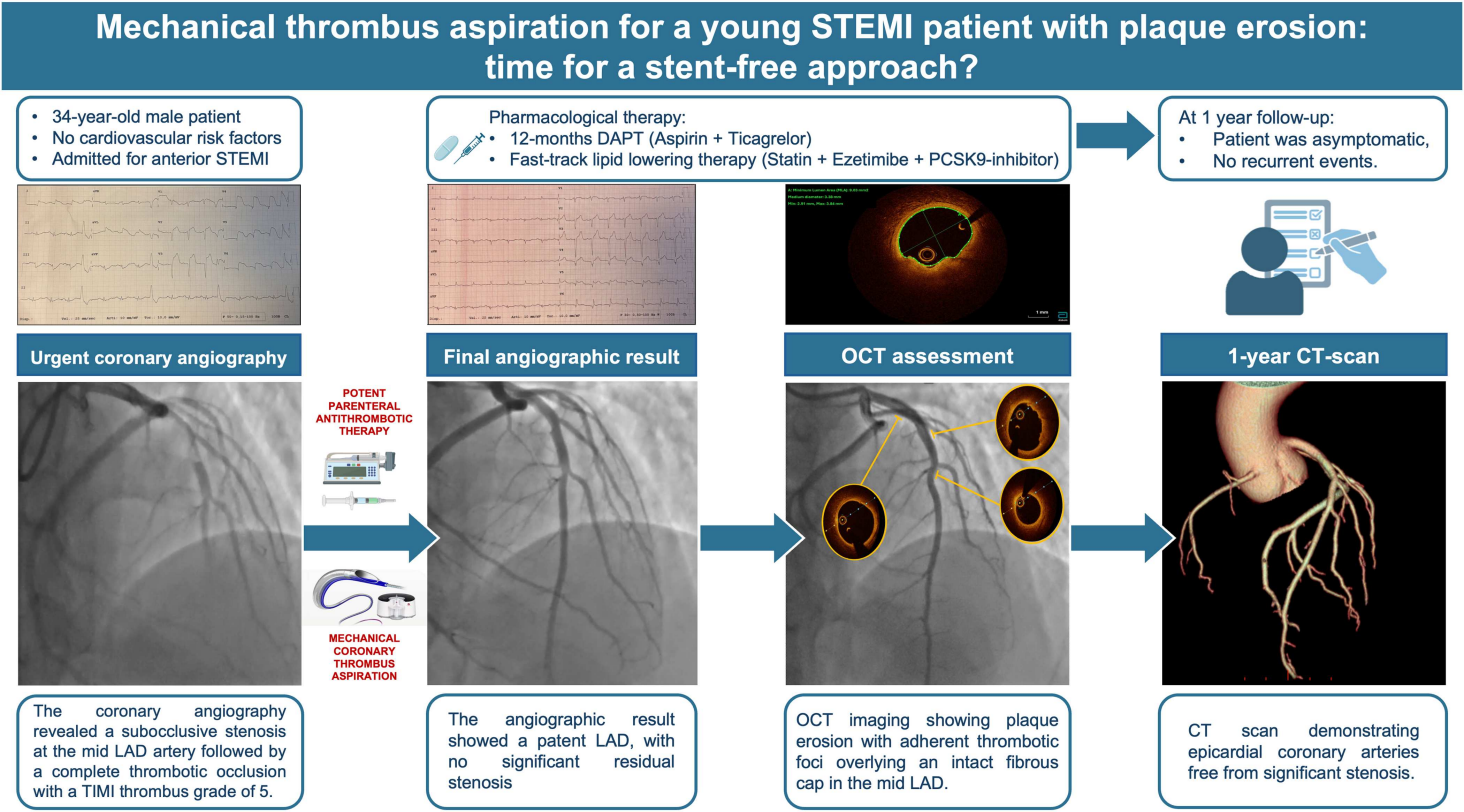
Overview of the case report with angiographic and intracoronary imaging evaluations along with the management strategy. [Color figure can be viewed at wileyonlinelibrary.com]

## Funding

The authors have nothing to report.

## Consent

Written informed consent was obtained from the patient for publication of clinical details and images.

## Conflicts of Interest

The authors declare no conflicts of interest.

## Data Availability

The data that support the findings of this study are available on request from the corresponding author. The data are not publicly available due to privacy or ethical restrictions.

## References

[1] H. Jia , F. Abtahian , A. D. Aguirre , et al., “In Vivo Diagnosis of Plaque Erosion and Calcified Nodule in Patients With Acute Coronary Syndrome by Intravascular Optical Coherence Tomography,” Journal of the American College of Cardiology 62, no. 19 (2013): 1748–1758, 10.1016/j.jacc.2013.05.071. 23810884 PMC3874870

[2] A. C. Fahed and I. K. Jang , “Plaque Erosion and Acute Coronary Syndromes: Phenotype, Molecular Characteristics and Future Directions,” Nature Reviews Cardiology 18, no. 10 (2021): 724–734, 10.1038/s41569-021-00542-3. 33953381

[3] A. Maino and F. A. Jaffer , “Outcomes Following Plaque Erosion‐Based Acute Coronary Syndromes Treated Without Stenting: The Plaque Matters,” Journal of the American Heart Association 11, no. 24 (December 2022): e028184, 10.1161/JAHA.122.028184. 36533624 PMC9798812

[4] R. Tian , R. Liu , J. Zhang , et al., “Efficacy and Safety of Intracoronary Versus Intravenous Tirofiban in Patients With ST‐Segment Elevation Myocardial Infarction Undergoing Primary Percutaneous Coronary Intervention: A Meta‐Analysis of Randomized Controlled Trials,” Heliyon 9, no. 5 (2023): e15842, 10.1016/j.heliyon.2023.e15842. 37180928 PMC10172923

[5] R. A. Byrne , X. Rossello , J. J. Coughlan , et al., “2023 ESC Guidelines for the Management of Acute Coronary Syndromes,” European Heart Journal 44, no. 38 (2023): 3720–3826, 10.1093/eurheartj/ehad191. 37622654

[6] F. Mach , K. C. Koskinas , J. E. Roeters van Lennep , et al., “2025 Focused Update of the 2019 ESC/EAS Guidelines for the Management of Dyslipidaemias,” European Heart Journal 46, no. 42 (2025): 4359–4378, 10.1093/eurheartj/ehaf190. 40878289

[7] P. Gargiulo , C. Basile , G. Galasso , et al., “Strike Early‐Strike Strong Lipid‐Lowering Strategy With Proprotein Convertase Subtilisin/Kexin Type 9 Inhibitors in Acute Coronary Syndrome Patients: Real‐World Evidence from the AT‐TARGET‐IT Registry,” European Journal of Preventive Cardiology 31, no. 15 (2024): 1806–1816, 10.1093/eurjpc/zwae170. 38788773

[8] L. Xing , E. Yamamoto , T. Sugiyama , et al., “EROSION Study (Effective Anti‐Thrombotic Therapy Without Stenting: Intravascular Optical Coherence Tomography‐Based Management in Plaque Erosion): A 1‐Year Follow‐Up Report,” Circulation: Cardiovascular Interventions 10, no. 12 (2017): e005860, 10.1161/CIRCINTERVENTIONS.117.005860. 29246916

[9] T. Svilaas , P. J. Vlaar , I. C. van der Horst , et al., “Thrombus Aspiration During Primary Percutaneous Coronary Intervention,” New England Journal of Medicine 358, no. 6 (2008): 557–567, 10.1056/NEJMoa0706416. 18256391

[10] O. Fröbert , B. Lagerqvist , G. K. Olivecrona , et al., “Thrombus Aspiration During ST‐Segment Elevation Myocardial Infarction,” New England Journal of Medicine 369, no. 17 (2013): 1587–1597, 10.1056/NEJMoa1308789. 23991656

[11] S. S. Jolly , J. A. Cairns , S. Yusuf , et al., “Randomized Trial of Primary PCI With or Without Routine Manual Thrombectomy,” New England Journal of Medicine 372, no. 15 (2015): 1389–1398, 10.1056/NEJMoa1415098. 25853743 PMC4995102

[12] S. S. Jolly , S. James , V. Džavík , et al., “Thrombus Aspiration in ST‐Segment‐Elevation Myocardial Infarction: An Individual Patient Meta‐Analysis: Thrombectomy Trialists Collaboration,” Circulation 135, no. 2 (2017): 143–152, 10.1161/CIRCULATIONAHA.116.025371. 27941066

